# Myelin alters the inflammatory phenotype of macrophages by activating PPARs

**DOI:** 10.1186/2051-5960-1-43

**Published:** 2013-08-02

**Authors:** Jeroen FJ Bogie, Winde Jorissen, Jo Mailleux, Philip G Nijland, Noam Zelcer, Tim Vanmierlo, Jack Van Horssen, Piet Stinissen, Niels Hellings, Jerome JA Hendriks

**Affiliations:** 1 Biomedical Research Institute, School of Life Sciences, Hasselt University / Transnational University Limburg, Diepenbeek, Belgium; 2 Department of Molecular Cell Biology and Immunology, VU University Medical Center, Amsterdam, The Netherlands; 3 Department of Medical Biochemistry, Amsterdam Medical Center, Amsterdam, The Netherlands

**Keywords:** Macrophages, Myelin, Multiple sclerosis, Phosphatidylserine, PPAR, Neuroinflammation

## Abstract

**Background:**

Foamy macrophages, containing myelin degradation products, are abundantly found in active multiple sclerosis (MS) lesions. Recent studies have described an altered phenotype of macrophages after myelin internalization. However, mechanisms by which myelin affects the phenotype of macrophages and how this phenotype influences lesion progression remain unclear.

**Results:**

We demonstrate that myelin as well as phosphatidylserine (PS), a phospholipid found in myelin, reduce nitric oxide production by macrophages through activation of peroxisome proliferator-activated receptor β/δ (PPARβ/δ). Furthermore, uptake of PS by macrophages, after intravenous injection of PS-containing liposomes (PSLs), suppresses the production of inflammatory mediators and ameliorates experimental autoimmune encephalomyelitis (EAE), an animal model of MS. The protective effect of PSLs in EAE animals is associated with a reduced immune cell infiltration into the central nervous system and decreased splenic cognate antigen specific proliferation. Interestingly, PPARβ/δ is activated in foamy macrophages in active MS lesions, indicating that myelin also activates PPARβ/δ in macrophages in the human brain.

**Conclusion:**

Our data show that myelin modulates the phenotype of macrophages by PPAR activation, which may subsequently dampen MS lesion progression. Moreover, our results suggest that myelin-derived PS mediates PPARβ/δ activation in macrophages after myelin uptake. The immunoregulatory impact of naturally-occurring myelin lipids may hold promise for future MS therapeutics.

## Background

Multiple sclerosis (MS) is characterized by central nervous system (CNS) infiltration of activated myelin-reactive lymphocytes and macrophages. Microglia and macrophages typically accumulate in the perivascular spaces and the brain parenchyma near terminal ovoids of transected axons [1]. Effector mechanisms of activated macrophages and microglia include internalization of myelin and secretion of inflammatory and toxic mediators, which negatively influence axonal and myelin integrity [2-7].

Macrophages are able to adopt divergent phenotypes depending on environmental cues [8-10]. In MS, macrophages and microglia initially display a pro-inflammatory phenotype [5-7,11]. However, upon internalization of myelin, they have been described to obtain anti-inflammatory characteristics [12-17]. We have previously demonstrated that myelin-derived cholesterol plays a role in directing this typical phenotype of myelin-phagocytosing macrophages by activating the sterol sensing liver-X-receptors (LXRs) [13]. Nonetheless, not all myelin-mediated effects on macrophages were induced by LXRs and cholesterol, and it is therefore likely that other myelin components also affect the phenotype of myelin-phagocytosing macrophages.

Phosphatidylserine (PS) is a phospholipid abundantly found in myelin [18]. One of the hallmarks of apoptosis is the translocation of PS to the outer membrane leaflet, where it serves as an “eat me” signal for phagocytic clearance [19,20]. Apoptotic cell clearance via PS skews macrophages and microglia towards an anti-inflammatory phenotype, similar to myelin-phagocytosing macrophages, hereby suppressing inflammation and maintaining homeostasis [21-24]. Since clearance of apoptotic cells by exposure of PS to macrophages and the subsequent induction of a tolerogenic phenotype has been associated with peroxisome proliferator-activated receptor (PPAR) activation [25], we determined whether a myelin-mediated PPAR activation is involved in directing the phenotype of macrophages during immune-mediated demyelination. Furthermore, we assessed the impact of PS-containing liposomes (PSLs) on neuroinflammation. We demonstrate that myelin as well as PS suppress the production of the inflammatory mediator nitric oxide (NO) by macrophages through activation of PPARβ/δ. Importantly, we provide compelling evidence that PSLs are immunosuppressive in an experimental MS animal model and that PPARβ/δ responsive genes and their corresponding proteins are markedly upregulated in myelin-phagocytosing macrophages in active demyelinating MS lesions. Taken together, our findings indicate that a myelin-mediated PPAR activation in macrophages may affect lesion progression in demyelinating diseases such as MS.

## Results

### Myelin and PS modulate the macrophages phenotype by activating PPARs

**Figure 1:**
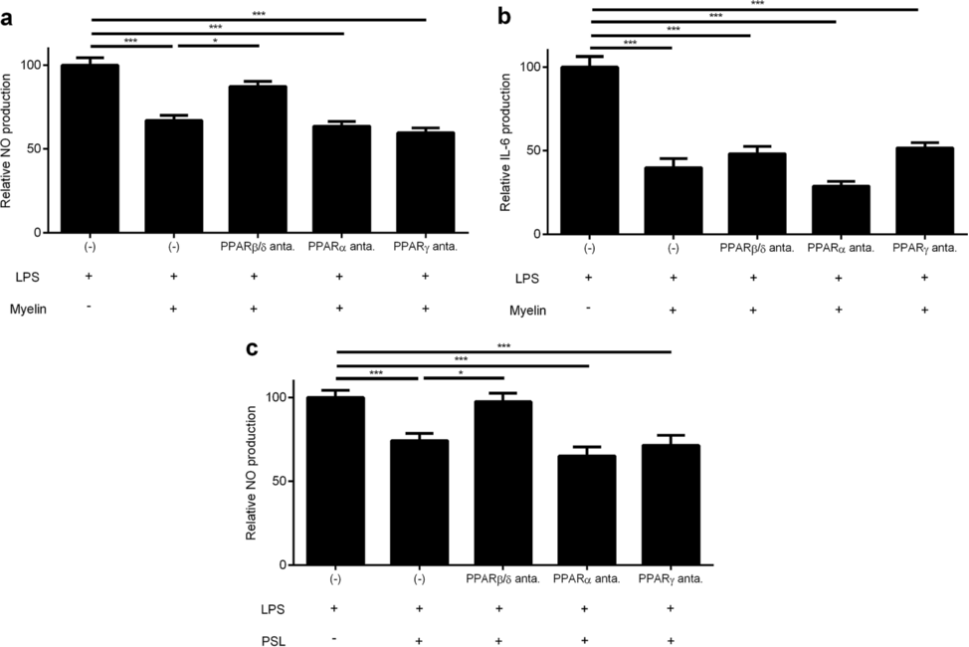
**Myelin and PSLs modulates the macrophages phenotype by PPARβ/δ activation. (a-c)** Relative NO and IL-6 concentration in supernatants of LPS stimulated control, myelin- or PSL-treated macrophages. Prior to administration of myelin or PSLs, cells were treated with antagonists for PPARα (GW6471), PPARβ/δ (GSK0660) or PPARγ (GW9662). The relative NO and IL-6 production is defined as the production of NO in experimental cultures divided by values in stimulated control cultures. Data represent the mean ± SEM of five independent experiments. (−): control cultures in which no antagonists were added.

To assess whether myelin affects the inflammatory phenotype of macrophages through activation of PPARα, β/δ or γ, macrophages were treated for 2 h with specific antagonists for PPARα (GW6471), β/δ (GSK0660) and γ (GW9662), prior to administration of myelin. While PPARα or PPARγ antagonists did not influence the reduced production of the inflammatory mediator NO by myelin-phagocytosing macrophages, a PPARβ/δ selective antagonist counteracted the decline in NO production (Figure 1a). The decrease in IL-6 production by myelin-phagocytosing macrophages was not affected by the antagonists (Figure 1b). This is in accordance with our previous study in which we demonstrated that suppression of IL-6 production by macrophages upon myelin internalization is LXRβ dependent. Notably, although macrophages expressed all PPAR subtypes, PPARβ/δ showed the highest expression (Additional file 1: Figure S1a).

To determine the involvement of PS in modulating the phenotype of macrophages upon myelin uptake, macrophages were incubated with PSLs and non-PS-containing liposomes (PCLs). PSLs have been described to mimic the functional effects of apoptotic cell clearance by macrophages [19,23]. First, the abundance of PS in isolated myelin was determined and compared to that in PSLs and PCLs. Flow cytometric analysis demonstrated that isolated myelin and PSLs contained similar levels of PS (Additional file 1: Figure S1b). Subsequently, the capacity of macrophages to internalize liposomes was determined. Like DiI-labeled myelin [12], both DiI-labeled PSLs and PCLs were internalized efficiently by macrophages *in vitro* (Additional file 1: Figure S1c). Finally, we assessed whether PS uptake affects the production of NO by macrophages through activation of PPARβ/δ. Similar to myelin-phagocytosing macrophages, the PPARβ/δ selective antagonist counteracted the reduced secretion of NO by PSL-treated macrophages (Figure 1c). In contrast to PSLs, PCLs did not alter NO production by macrophages (Additional file 1: Figure S1d). Of note, the PPARβ/δ antagonist did not affect the capacity of macrophages to internalize myelin or liposomes (Additional file 1: Figure S1e-g), indicating that a reduced internalization of myelin and liposomes does not account for the increase in NO production following administration of the PPARβ/δ antagonist. These results show that myelin modulates the inflammatory phenotype of macrophages by activating PPARβ/δ and suggest that PS in myelin is responsible for this activation.

### Systemically administered liposomes home primarily to splenic macrophages and ameliorate EAE

**Figure 2:**
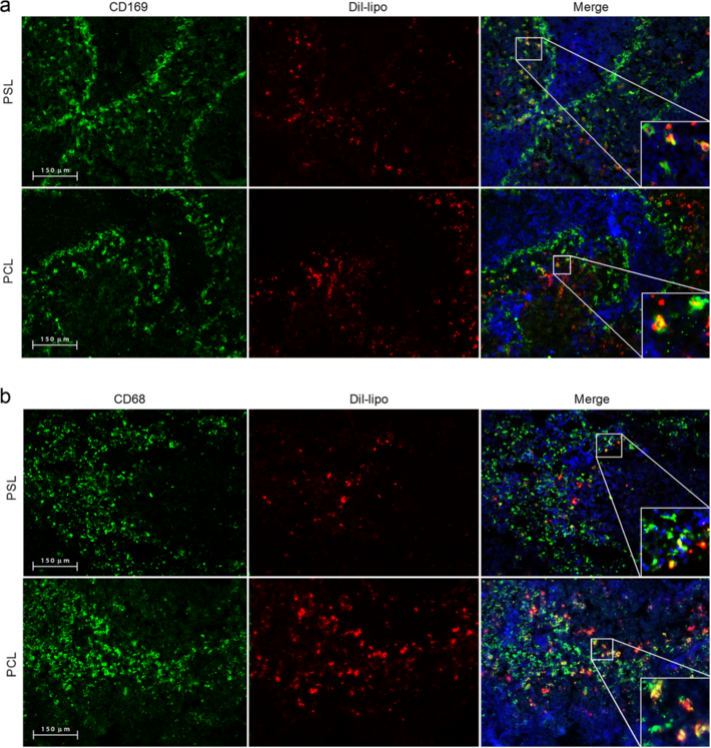
**Systemically administered liposomes home primarily to splenic marginal zone and red pulp macrophages. (a,b)** Healthy rats were injected with 5 mg/kg DiI-labeled PCLs and PSLs. Splenic cryosections were stained with CD169 (**a**, marginal metallophilic and marginal zone macrophages) and CD68 (**b**, red pulp macrophages). One representative experiment is shown (20× magnification).

To determine if PS uptake by macrophages influences the pathology and severity of experimental autoimmune encephalomyelitis (EAE), immunized rats were treated with PBS, PCLs or PSLs. First, the homing properties of liposomes after intravenous administration of DiI-labeled PSLs were determined by flow cytometry and immunohistochemistry. In healthy animals, injected PSLs were primarily retrieved in the spleen and liver (Additional file 2: Figure S2a). Furthermore, immunohistochemical analysis demonstrated that especially splenic CD169^+^ marginal zone and CD68^+^ red pulp macrophages contained liposomes (Figure 2a and b). The absence of liposomes in CNS tissue suggests that liposomes are incapable of crossing an intact blood–brain-barrier. Similar to healthy animals, PSLs homed primarily to the spleen and liver when injected after EAE onset (Additional file 2: Figure S2b). However, in these immunized animals we were also able to detect sporadic CD68^+^ macrophages in the spinal cord containing DiI-liposomes (Additional file 2: Figure S2c). The high number of liposomes present in the lungs may be explained by the fact that they are trapped in the narrow capillaries of the lung. Collectively, these results show that PSLs migrate towards splenic red pulp and marginal zone macrophages after systemic administration, but can also enter the CNS during EAE. Whether liposomes are phagocytosed by systemic macrophages that subsequently enter the CNS or freely cross the compromised blood–brain-barrier remains to be clarified.

**Figure 3:**
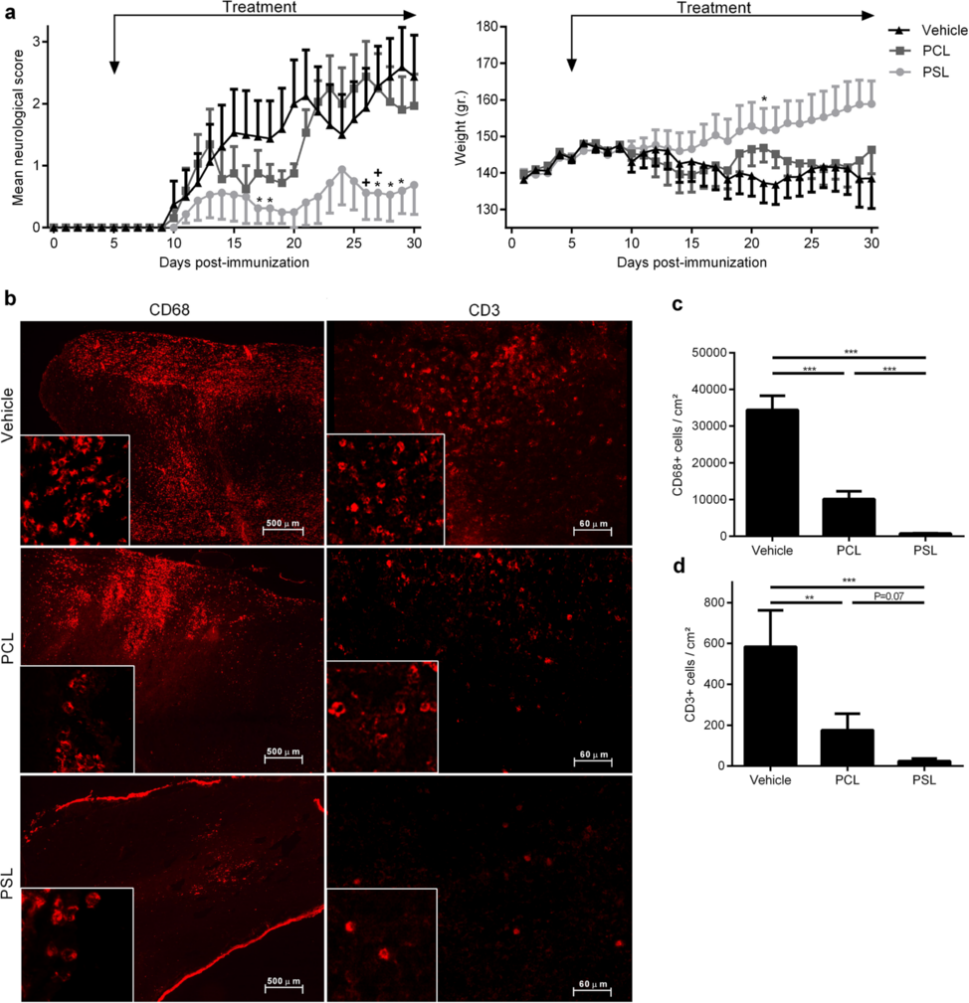
**Intravenously injected PSLs reduce CNS infiltration of immune cells and ameliorate EAE. (a)** MOG-immunized animals were treated daily with PBS (n=8; black), 5 mg/kg PCLs (n=8; dark grey) or 5 mg/kg PSLs (n=8; light grey) starting from day 5. Neurological score and weight were assessed daily. Data represent the mean ± SEM. ^*^P < 0.05 (vehicle vs PSL), ^+^P < 0.05 (PCL vs. PSL). **(b)** Spinal cord tissue was isolated 30 dpi and stained with CD3 (20× magnification) and CD68 (4× magnification). One representative image is shown. **(c,d)** Quantification of T cell and macrophage infiltration in spinal cord tissue 30 dpi. Nine cryosections, covering the complete length of the spinal cord, were stained with CD68 **(c)** and CD3 **(d)**. A 4× magnification was used to determine the amount of immune cell infiltration. Data represent the mean ± SEM of 4 animals.

To assess the effect of PSLs on EAE development, immunized animals were treated daily with PSLs, PCLs or PBS, starting 5 days post-immunization (dpi). PSL-treated animals displayed a significantly reduced neurological score compared to PCL- (area under the curve (AUC), PSL: 7.41 ± 4.38 vs PCL: 30.13 ± 6.11, P < 0.05, Figure 3a) and vehicle-treated animals (AUC, PSL: 7.41 ± 4.38 vs vehicle: 34.53 ± 10.80, P < 0.05, Figure 3a). Furthermore, disease incidence was lower in animals treated with PSLs (62.5%), compared to PCL- (100%) and vehicle-treated animals (87.5%). The reduced disease severity in PSL-treated animals was paralleled with decreased numbers of CNS infiltrating macrophages and T cells (Figure 3b-d). Although PCL treatment did not significantly affect disease severity, PCL-treated animals did have significantly reduced numbers of infiltrated immune cells in the CNS, as compared to vehicle treated animals.

**Figure 4:**
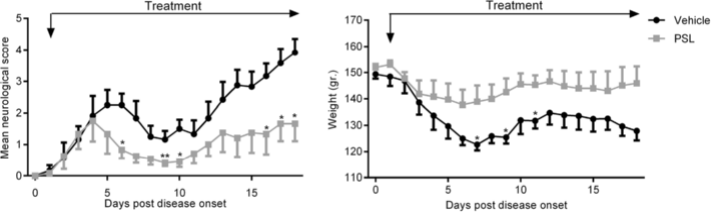
**PSLs have a therapeutic effect on EAE.** MOG-immunized animals were treated daily with PBS (n=6; black) or 5 mg/kg PSLs (n=6; light grey) starting at disease onset. Neurological score and weight was assessed daily. Data represent the mean ± SEM.

To determine the therapeutic potential of PSLs, EAE animals were treated daily with PSLs or PBS, starting one day after disease onset. Similar as in the prophylactic regimen, PSL-treated animals displayed a significantly reduced neurological score compared to vehicle-treated animals (AUC, PSL: 17.5 ± 5.21 vs vehicle: 34.25 ± 3.53, P < 0.05, Figure 4). Collectively, these data show that PSLs attenuate the course of EAE when administered both before and after disease onset.

### PSLs modulate T cell proliferation and the expression of pro- and anti-inflammatory mediators in the spleen

**Figure 5:**
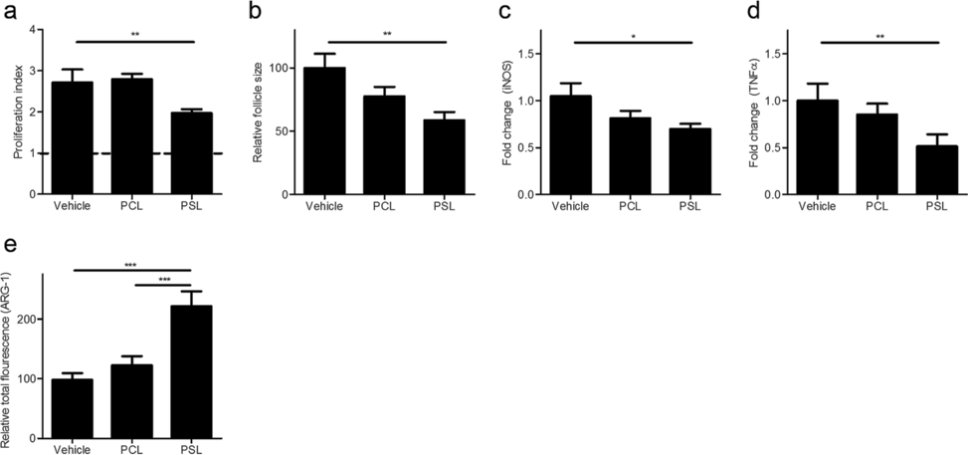
**PSLs affect splenic cognate antigen specific proliferation and expression of TNFα, iNOS and ARG-1. (a)** Cognate antigen specific proliferation (10 dpi) of splenic cultures was assessed by culturing splenic cells from vehicle, PCL and PSL treated animals with MOG. Proliferation was assessed by [3H]thymidine incorporation. Non-stimulated cultures were used as control (dotted line). Data represent the mean ± SEM of four experiments. **(b)** The size of the splenic white pulp was determined using ImageJ software. Three cryosections per animal were stained with CD200R, a marker for myeloid cells, after which the surface area surrounded by the marginal metallophilic macrophages was determined. Five images (4× magnification) per section were taken to calculate the mean white pulp size. Data represent the mean ± SEM of four animals. **(c,d)** Comparison of fold changes between vehicle, PCL and PSL-treated spleens 10 dpi. Relative quantification of iNOS **(c)** and TNFα **(d)** was accomplished by using the comparative C_t_ method. Data were normalized to the most stable reference genes, determined by Genorm (Pgk1 and Rpl13a). Data represent the mean ± SEM of 4 experiments. **(e)** Spleen cryosections were stained with ARG-1 after which the total corrected fluorescence was determined using ImageJ software, as described previously [65]. Three cryosections were stained and 6 images (10× magnification) were taken per section. Data represent the mean ± SEM of four animals.

To determine the impact of PSLs on T cell proliferation, cognate antigen specific proliferation of splenic cultures from vehicle-, PCL- and PSL-treated animals was assessed. Splenic T cells from PSL-treated animals showed a significantly reduced myelin oligodendrocyte glycoprotein (MOG) reactivity, compared to both vehicle- and PCL-treated animals (Figure 5a). In line with this, the mean white pulp surface area in the spleen, determined by measuring the marginal metallophilic macrophages-surrounded area, was reduced in animals treated with PSLs (Figure 5b). Representative images of these measurements are depicted in Additional file 3: Figure S3a. No differences in splenic gene expression of transcription factors characteristic for divergent T cell subsets, such as T-bet (Th1), GATA-3 (Th2), RORγt (Th17) and Foxp3 (Treg), were detected (Additional file 3: Figure S3b).

To further determine the underlying mechanisms by which PSLs modulate EAE pathogenesis, splenic expression of inducible nitric oxide synthase (iNOS), TNFα and arginase-1 (ARG-1) was assessed. Whereas iNOS and TNFα are typical inflammatory mediators produced by macrophages, ARG-1 is a commonly used marker for alternatively activated macrophages. Reduced iNOS and TNFα mRNA levels were observed in spleens of PSL-treated animals (Figure 5c and d). Furthermore, although splenic ARG-1 mRNA expression was unaffected, the total fluorescent intensity of splenic ARG-1 expression was significantly increased in animals treated with PSLs, indicating enhanced arginase activity (Figure 5e). Representative images of these measurements are depicted in Additional file 3: Figure S3c. The altered expression of iNOS, TNFα and ARG-1 in PSL-treated animals is in agreement with the ability of PSLs to affect the expression of these mediators by macrophages *in vitro* (Figure 1c, Additional file 3: Figure S3d and S3e).

These results demonstrate that PSL treatment suppresses T cell proliferation without affecting their polarization. Furthermore, we show that PSLs affect the expression of iNOS, TNFα and ARG-1 *in vivo* in a similar manner as PSL treatment of macrophages *in vitro*. These immunosuppressive and anti-inflammatory properties of PSLs likely contribute to the observed reduction in neuroinflammation after PSL treatment.

### Myelin-phagocytosing macrophages display increased activation of PPARs in active MS lesions

**Figure 6:**
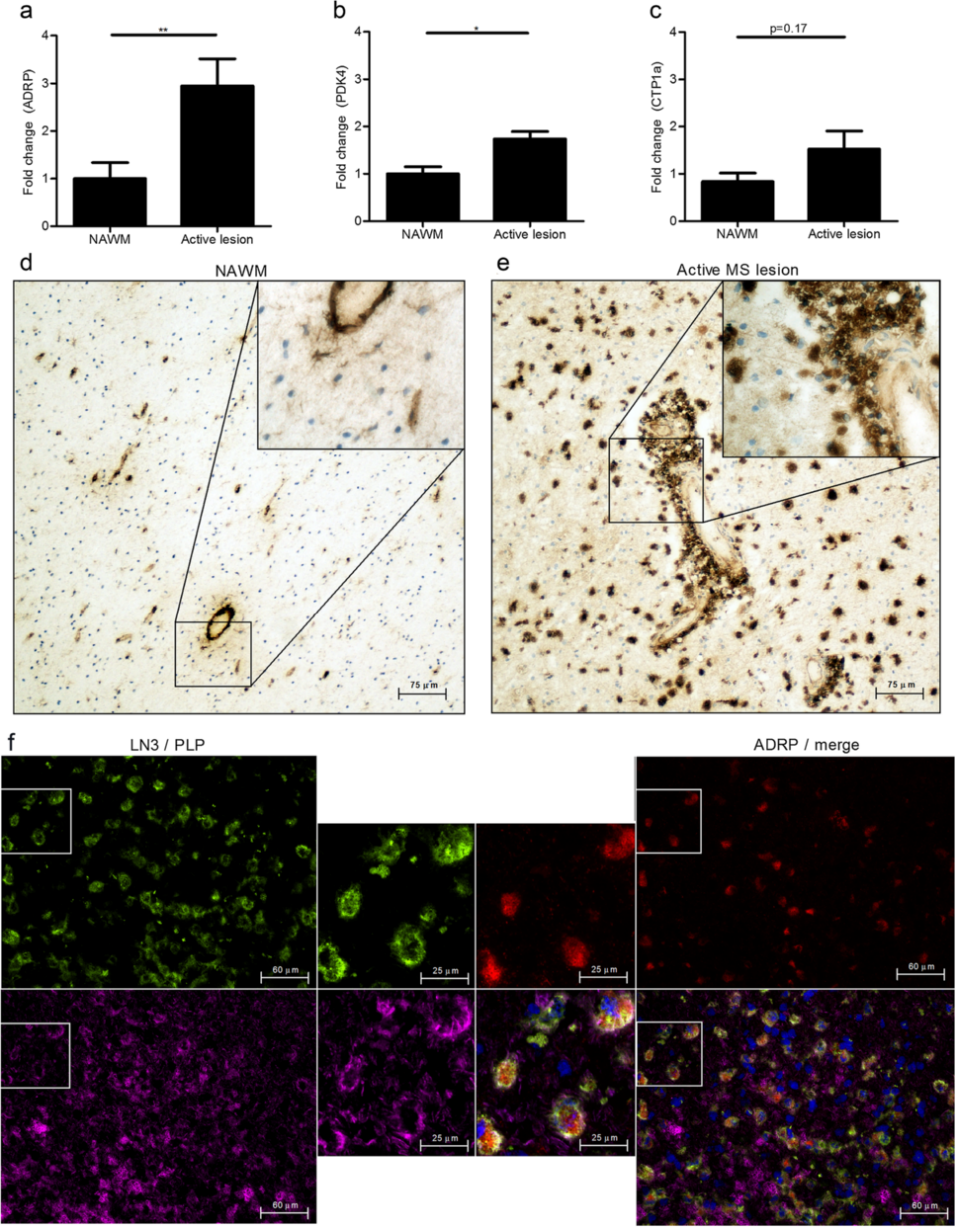
**PPARs are activated in myelin-phagocytosing macrophages during active MS. (a-c)** Comparison of fold changes between non-demented controls (n=5) and active MS lesions (n=5). Relative quantification of ADRP **(a)**, PDK4 **(b)** and CTP1a **(c)** was accomplished by using the comparative C_t_ method. Data were normalized to the most stable reference genes, determined by Genorm (YHWAZ and Rpl13a). **(d,e)** Normal-appearing white matter **(d)** and an active MS lesion **(e)** stained for ADRP. One representative image is shown (20× magnification). **(f)** Active MS lesion stained with HLA-DR (green top; left corner), ADRP (red; top right corner), PLP (magenta; bottom left corner) and DAPI (blue, bottom right corner). One representative image is shown (40× magnification).

To elucidate whether PPARs are also active in myelin-containing macrophages in MS lesions, we determined PPARβ/δ activation in MS CNS tissue by quantitative PCR and immunohistochemistry. The expression of PPARβ/δ responsive genes adipose differentiation related protein (ADRP), carnitine palmitoyltransferase I (CPT1a) and pyruvate dehydrogenase kinase isozyme 4 (PDK4) was assessed [26-28]. RNA was isolated from regions accommodating lipid-containing macrophages and microglia, determined by Oil Red O staining. Expression of ADRP and CTP1a mRNA was increased in active MS lesions, compared to non-demented controls (Figure 6a-c). To establish whether PPARβ/δ responsive genes are induced in myelin-containing macrophages in MS lesions, the expression of ADRP was determined by immunohistochemistry. In agreement with the PCR data, immunohistochemical analysis showed that ADRP was highly abundant in active MS lesions compared to the surrounding normal-appearing white matter (Figure 6d and e). Moreover, macrophages containing myelin were intensely stained by anti-ADRP in active MS lesions (Figure 6F). Semi-quantitative analysis demonstrated that 60% of the HLA-DR^+^ macrophages co-expressed ADRP. Furthermore, ADRP was exclusively expressed by HLA-DR^+^ macrophages and >95% of ADRP^+^HLA-DR^+^ macrophages contained myelin. These data show that myelin-phagocytosing macrophages in MS lesions have active PPARβ/δ signaling.

## Discussion

In this study we aimed to determine whether myelin directs the inflammatory phenotype of macrophages by PPAR activation and how this phenotype impacts lesion progression in MS. We show that internalization of myelin and PSLs inhibit NO production by macrophages through activation of PPARβ/δ. Furthermore, we demonstrate that PSLs, internalized by splenic macrophages, significantly reduce clinical signs in an experimental MS animal model by suppressing autoaggressive T cells, lowering the expression of inflammatory mediators and inhibiting infiltration of immune cells into the CNS. Interestingly, PPARβ/δ-responsive genes and their corresponding proteins were markedly increased in myelin-containing macrophages during active demyelination in MS. Collectively, these findings indicate that myelin modulates the inflammatory phenotype of macrophages by activating PPARβ/δ and suggest that PS in myelin is responsible for this activation. The myelin-mediated activation of PPARs in macrophages may dampen lesion progression and explain the relapse-remitting nature of MS.

Myelin contains various lipids that may modify the functional properties of macrophages. Recently, we demonstrated that myelin-derived cholesterol influences the phenotype of macrophages through activation of LXRs. While the suppressed IL-6 production by myelin-phagocytosing macrophages was LXRβ dependent, the observed reduction in NO production was unaffected in LXR-deficient macrophages. PS is a constituent of myelin and a potent regulator of inflammatory responses. *In vitro*, clearance of apoptotic cells and PSLs skews macrophages towards a tolerogenic phenotype [21,23,29-35]. Likewise, myelin internalization induces an anti-inflammatory, immunosuppressive phenotype in macrophages [12-17]. Here we show that both myelin and PSLs lower NO production by macrophages. Moreover, we demonstrate that PPARβ/δ activation underlies the effect that PSLs and myelin have on the phenotype of macrophages. The myelin-mediated activation of PPARβ/δ corresponds with the fact that myelin-phagocytosing macrophages have an upregulated expression of genes involved in PPAR signalling [13]. Moreover, these findings are in line with studies showing that PPARβ/δ is a transcriptional sensor of apoptotic cells and that it regulates the program of alternative activation in macrophages [25,36-39]. Interestingly, in a carrageenan-induced mouse paw edema model it has been shown that PSLs are capable of suppressing inflammation *in vivo* by activating PPARγ [40], indicating that PSLs can affect inflammation via multiple PPAR subtypes.

We demonstrate that systemically administered PSLs, primarily internalized by splenic CD68^+^ red pulp and CD169^+^ marginal zone macrophages, suppress EAE in both prophylactic and therapeutic settings. In line with our findings, other studies demonstrated that administration of non-encapsulated PS ameliorates EAE when administered before or after disease onset [41,42]. In these studies it was described that the beneficial effect of PS was mediated by a direct effect of PS on autoaggressive T cell responses. Similar, PSLs have been described to modulate T cell differentiation and suppress antigen-specific immune responses *in vivo*[22,43]. We now provide evidence that PS not only affects T cell responses but also influences macrophage behavior. The PS-mediated change of the macrophage phenotype will contribute to the immunosuppressive capacity of PSLs. *In vivo*, PSLs have been described to promote the resolution of inflammation by modulating macrophage function in a model for inflammatory bone loss and myocardial infarction [31,33]. As ARG-1 activity suppresses antigen-specific T cell responses [44,45], the increased splenic expression of ARG-1 in PSL-treated animals may account for the observed inhibition of splenic T cell proliferation in our model. In addition to the immunosuppressive effects of PSLs, we observed a marked reduction in the numbers of macrophages and T cells infiltrating into the CNS of PSL-treated EAE animals. This indicates that PSLs influence immune cell trafficking towards the CNS, in addition to or as a result of modulating the macrophages phenotype or T cell proliferation. In summary, results from our study indicate that PSLs will affect neuroinflammation by modulating the functional properties of macrophages.

Interestingly, we demonstrate that the expression of PPARβ/δ responsive genes and proteins is upregulated in active MS lesions, especially in myelin-phagocytosing macrophages. All PPAR subtypes have been described to regulate the differentiation of macrophages towards an anti-inflammatory phenotype [36-39,46-48]. Moreover, agonists for all PPARs reduce CNS inflammation and demyelination in EAE [49-56]. The importance of PPARβ/δ signaling in maintaining immune-homeostasis and preventing systemic autoimmunity is illustrated by the fact that macrophage-specific PPARβ/δ deficiency delays clearance of apoptotic cells and increases autoantibody production [25]. Our finding that PPARβ/δ is active in myelin-containing macrophages in active MS lesions indicates that degraded myelin also activates PPARβ/δ in macrophages in the human brain. This myelin-mediated PPAR activation may affect lesion progression by inducing an anti-inflammatory environment and by influencing the activity of infiltrating T cells. Moreover, as PPARβ/δ activation enhances the internalization of apoptotic cells [25], myelin-mediated PPARβ/δ activation may promote clearance of myelin debris, which inhibits oligodendrocyte precursor maturation and axonal regeneration [57-60], thereby stimulating repair.

## Conclusion

This report provides an interesting link between demyelination, lipid metabolism and macrophage-mediated inflammation. Our data indicate that myelin modulates the inflammatory phenotype of macrophages by activating PPARβ/δ and suggests that PS in myelin is responsible for this activation. Since PSLs ameliorate EAE and PPARβ/δ is activated in myelin-phagocytosing macrophages in active MS lesions, we hypothesize that during MS pathogenesis myelin uptake by macrophages induces naturally-occurring regulatory mechanisms by PPAR activation. The identification of myelin-derived lipids capable of dampening macrophage-mediated inflammation can potentially explain the relapse-remitting nature of MS and holds promise for future intervention strategies aimed at reducing neuroinflammation in disorders like MS.

## Methods

### Animals

Female Dark Agouti rats, 8–10 weeks old, were purchased from Harlan Netherlands B.V. (Horst, The Netherlands). Animals were housed in the animal facility of the Biomedical Research Institute of Hasselt University. Experiments were conducted in accordance with institutional guidelines and approved by the Ethical Committee for Animal Experiments of Hasselt University.

### Myelin isolation

Myelin was purified from rat brain tissue by means of density-gradient centrifugation, as described previously [61]. Myelin protein concentration was determined by using the BCA protein assay kit (Thermo Fisher Scientific, Erembodegem, Belgium). LPS content was determined using the Chromogenic Limulus Amebocyte Lysate assay kit (Genscript Incorporation, Aachen, Germany). Isolated myelin contained a neglectable amount of endotoxin (<1.8×10^-3^ pg/μg myelin). Expression of phosphatidylserine on myelin, PSLs and PCLs was determined by flow cytometry using FITC-labeled Annexin V (Biolegend, Antwerpen, Belgium).

### Preparation of liposomes

Liposomes were prepared as described previously [62]. In brief, nitrogen-dried lipid films containing various phospholipids were suspended in PBS and sonicated for 10 min on ice. The liposomes were composed of either phosphatidylcholine (PC; Sigma-Aldrich, Bornem, Belgium) only or a combination of PC and PS (Sigma-Aldrich) at a molar ratio of 7:3. In some experiments, liposomes were fluorescently labeled with 1,1”-diotadecyl-3,3,3’,3’,-tetramethylindocarbocyanide perchlorate (DiI; Sigma-Aldrich). For this, liposomes were incubated with DiI for 10 min at 37°C, after which liposomes were centrifuged to remove non-encapsulated DiI. Flow cytometry was used to assess labeling efficacy and the degree of DiI-liposome uptake.

### Cell culture

Rat macrophages (NR8383; ATCC, Molsheim, France) were cultured in RPMI 1640 medium (Lonza, Vervier, Belgium) enriched with 10% fetal calf serum (Hyclone, Erenbodegen, Belgium), 50 U/ml penicillin and 50 U/ml streptomycin (Invitrogen, Merelbeke, Belgium). Cells were treated for 24 h with 100 μg/ml myelin, 250 μg/ml PSLs or 250 μg/ml PCLs in 96-well plates (15 × 10^4^ cells/well). Subsequently, cells were stimulated with 100 ng/ml LPS (Sigma-Aldrich) for 9 h for RNA isolation or 18 h for analysis of culture supernatants. To evaluate the involvement of PPARs, macrophages were pretreated for 2 h with antagonists for PPARα (GW6471, 10 μM, dissolved in DMSO), PPARβ/δ (GSK0660 10 μM, dissolved in DMSO) and PPARγ (GW9662, 1 μM, dissolved in DMSO) (all from Sigma-Aldrich). Cell viability was determined using a 3-(4,5-Dimethylthiazol-2-yl)-2,5-diphenyltetrazolium bromide assay (MTT assay). In short, following LPS stimulation the medium was aspirated and replaced by medium supplemented with 12,5 μl sterile filtered MTT (Sigma-Aldrich). After 4h incubation, the unreacted dye was aspirated and the insoluble formazan crystals were dissolved in 175 μl of a DMSO – glycine solution. Absorbance was measured at 540–550 nm.

### Nitrite formation and cytokine production

Culture supernatants of macrophages were collected after 18 h stimulation with LPS. Release of NO was determined using a Griess reagent system (Promega, Leuven, Belgium). Cytokine concentrations in culture supernatants were determined using a rat TNFα (Ebioscience, Vienna, Austria) and rat IL-6 ELISA (R&D systems, Abingdon, UK).

### Induction of EAE and systemic liposome treatment

Rats were immunized subcutaneously at the base of the tail with 140 μg of recombinant human MOG emulsified in incomplete Freund’s adjuvant (Sigma-Aldrich) supplemented with 500 μg of heat-inactivated Mycobacterium tuberculosis (Difco, Detroit, USA). Immunized animals were treated daily with PBS, 5 mg/kg PCLs or 5 mg/kg PSLs beginning 5 dpi or at disease onset. A total of 400 μl, containing liposomes or PBS, was injected intravenously in the tail vein. In parallel, to track liposomes in healthy and immunized animals, rats were injected with 5 mg/ml of DiI-labeled liposomes and sacrificed after 24 h. Immunized rats were weighed and scored daily according to the following neurological scale: 0.5 = partial loss of tail tonus, 1 = complete loss of tail tonus, 2 = hind limb paresis, 3 = hind limb paralysis, 4 = moribund, 5 = death.

### [^3^H]Thymidine incorporation

Cognate antigen specific proliferation of T cells was determined by measuring the amount of [^3^H]thymidine incorporation. In short, ficoll-separated splenic cells (20 × 10^4^, isolated 10 dpi) were cultured in RPMI 1640 medium supplemented with 50 U/ml penicillin, 50 U/ml streptomycin, 20 μM 2-mercapto-ethanol (Sigma-Aldrich), 1% sodium pyruvate (Invitrogen), 1% MEM non-essential amino acids (Invitrogen), 2% deactivated autologous serum and 20 μg/ml MOG. After 48 h, 1 μCi [^3^H]thymidine (Amersham, Buckinghamshire, UK) was added to the culture for 18 h. Next, cells were harvested with an automatic cell harvester (Pharmacia, Uppsala, Sweden) and radioactivity was measured in a β-plate liquid scintillation counter (Wallac, Turku, Finland).

### Quantitative PCR

Total RNA from cultures and tissues was prepared using the RNeasy mini kit or RNeasy lipid tissue mini kit (Qiagen, Venlo, The Netherlands), according to the manufacturer’s instructions. The RNA concentration and quality was determined with a NanoDrop spectrophotometer (Isogen Life Science, IJsselstein, The Netherlands). RNA was converted to cDNA using the reverse transcription system (Promega) and quantitative PCR was subsequently conducted on a StepOnePlus detection system (Applied biosystems, Gaasbeek, Belgium), as previously described [13]. Relative quantification of gene expression was accomplished by using the comparative C_t_ method. Data were normalized to the most stable reference genes [63,64]. Primers were chosen according to literature or designed using Primer-Express (http://www.ncbi.nlm.nih.gov/tools/primer-blast). Details of the primers used are shown in Additional file 4: Table S1 and clinical data of MS patients and non-neurological controls (The Netherlands Brain Bank) are depicted in Additional file 5: Table S2.

### Immunohistochemistry

Animals were sacrificed in the effector (10 dpi) and chronic phase (30 dpi) of EAE, after which brains, spinal cords and spleens were isolated and snap-frozen. Frozen brain material from MS patients and non-demented controls was obtained from the Netherlands Brain Bank (NBB, Amsterdam, Netherlands). Material was sectioned with a Leica CM1900UV cryostat (Leica Microsystems, Wetzlar, Germany) to obtain 7 μm slices. The extent of immune cell infiltration in spinal cord sections was determined using monoclonal mouse anti-rat CD68 (ED1 clone, AbD Serotec) and monoclonal mouse anti-rat CD3 (1F4 clone, AbD Serotec). For tracking of DiI-liposomes, spleen and spinal cord cryosections were stained with anti-rat CD68 and monoclonal mouse-anti-rat CD169 (ED3 clone, AbD serotec). Additionally, splenic sections were stained with monoclonal mouse anti-Arginase 1 (19/Arginase I clone, BD Biosciences, Erembodegem, Belgium) and polyclonal goat anti-CD200R (M-21 clone, Santa Cruz, Heidelberg, Germany). Human brain material was stained with polyclonal rabbit anti-ADRP (Abcam, Cambridge, UK), monoclonal mouse anti-HLA-DR (LN3 clone, eBioscience) and polyclonal rabbit anti-PLP (Abcam). Alexa fluor secondary antibodies were all purchased from Invitrogen (Merelbeke, Belgium). In short, dried cryosections were fixed in acetone for 10 minutes, after which they were blocked for 30 minutes with 10% normal serum from the same species as the secondary antibody. Sections were incubated overnight with primary antibodies, secondary antibodies were added for 2 h. Nuclei were visualized using DAPI (Invitrogen). Control stainings were performed by omitting the primary antibody. PBS containing 0.05% Tween 20 (Merck, Darmstadt, Germany) was used for washing and diluting the antibodies. For 3, 3′ diaminobenzidine staining, the Dako Envision+ kit (Dako) was used according to manufacturer’s instructions. Sections were counterstained with hematoxylin (Merck, Darmstadt, Germany). Analysis was carried out using a Nikon eclipse 80i microscope and NIS Elements BR 3.10 software (Nikon, Tokyo, Japan).

### Statistical analysis

Data were statistically analyzed using GraphPad Prism for windows (version 4.03) and are reported as mean ± SEM. D’Agostino and Pearson omnibus normality test was used to test normal distribution. An ANOVA (post-hoc; Tukey) or two-tailed unpaired Student t-test was used for normally distributed data sets. The Kruskal-Wallis (Dunns post hoc comparison) or Mann–Whitney analysis was used for data sets which did not pass normality. EAE scores were analyzed using the Kruskal-Wallis (Dunns post hoc comparison) and Mann–Whitney analysis. An overall effect of treatment was assessed by measuring the AUC. *P < 0.05, **P < 0.01 and ***P < 0.001.

## Competing interests

The authors declare that they have no competing interests.

## Authors’ contributions

JB, JvH, NZ, PS, NH and JH conceived and designed the experiments. JB, WJ and JM performed the experiments. NZ, PN and TV provided scientific input. JB wrote the paper. WJ, JM, PN, NZ, TV, JvH, PS, NH and JH revised the manuscript. All Authors read and approved the final manuscript.

## Supplementary Material

Additional file 1: Figure S1 Characteristics of macrophages, myelin, liposomes and PPAR antagonists. **(a)** Comparison of PPARα, PPARβ/δ and PPARγ mRNA expression in NR8383 cells. Relative quantification of PPARα, β/δ and γ was accomplished by using the comparative C_t_ method. Data were normalized to the most stable reference genes, determined by Genorm (TBP and HMBS). Data represent the mean ± SEM of two independent experiments. **(b)** Annexin V staining of myelin, PSLs and PCLs. Unstained myelin and liposomes were used as control (grey area). One representative experiment is shown (n=2). **(c)** Internalization of PSLs and PCLs by macrophages. Macrophages were treated with DiI-labeled PCLs (DiI-PCL) and PSLs (DiI-PSL) for 1.5 h. Liposome uptake was assessed by flow cytometry. Data represent the mean ± SEM of two experiments. **(d)** Relative NO concentration in supernatants of LPS stimulated control and PCL-treated macrophages. The relative NO production is defined as the production of NO in experimental cultures divided by values in stimulated control cultures. Data represent the mean ± SEM of four independent experiments. **(e-g)** Internalization of myelin and liposomes by macrophages after pretreatment with PPAR antagonists. Macrophages were treated for 2 h with antagonists for PPARα (GW6471), PPARβ/δ (GSK0660) or PPARγ (GW9662) prior to administration of DiI-labeled myelin (DiI-Myelin), PSLs (DiI-PSLs) and PCLs (DiI-PCLs) for 1.5 h. Uptake was assessed by flow cytometry. The relative phagocytosis is defined as the phagocytosis of myelin or liposomes in experimental cultures divided by values in control conditions. Data represent the mean ± SEM of five independent experiments. (−): control cultures in which no antagonists were added. Click here for file

Additional file 2: Figure S2 Homing properties of liposomes following systemic administration. **(a, b)** Healthy **(a)** and immunized **(b)** rats were injected with 5 mg/kg DiI-labeled PSLs. Immunized animals received DiI-labeled liposomes one day after disease onset. After 24 h animals were sacrificed and the homing properties of liposomes were assessed by flow cytometry. To determine the homing of liposomes the amount of DiI+ cells (cells which internalized liposomes) was divided by the total number of organ cells analyzed. One representative experiment is shown (n=2). **(c)** Immunized rats were injected with 5 mg/kg DiI-labeled PSLs (DiI-PSL) at disease onset. After 24 h animals were sacrificed and spinal cord cryosections were stained with CD68 (40× magnification). One representative experiment is shown (n=3). Click here for file

Additional file 3: Figure S3 Impact of liposomes on the inflammatory properties of splenic tissue in EAE animals. **(a)** Spleen tissue was isolated 10 dpi and stained with CD200R (10× magnification). One representative image is shown. WP: white pulp. **(b)** Comparison of fold changes between vehicle, PCL and PSL-treated spleens 10 dpi. Relative quantification of T-bet, GATA-3, RORγt and Foxp3 was accomplished by using the comparative C_t_ method. Data were normalized to the most stable reference genes, determined by Genorm (Pgk1 and Rpl13a). Data represent the mean ± SEM of 4 animals. **(c)** Spleen tissue was isolated 10 dpi and stained with ARG-1 (10× magnification). An identical exposure time and gain was used for all images. One representative image is shown. **(d)** Relative TNFα concentration in supernatants of LPS stimulated control and PSL-treated macrophages. The relative TNFα production is defined as the production of TNFα in experimental cultures divided by values in stimulated control cultures. Data represent the mean ± SEM of five independent experiments. **(e)** Comparison of fold changes between LPS stimulated control and PSL-treated macrophages. Relative quantification of ARG-1 gene expression was accomplished by using the comparative C_t_ method. Data were normalized to the most stable reference genes, determined by Genorm (TBP and HMBS). Data represent the mean ± SEM of three independent experiments. Click here for file

Additional file 4: Table S1 Primer details. Click here for file

Additional file 5: Table S2 Clinical data of MS patients and non-neurological controls. Click here for file
