# Supplementary figures and images for: Myelin alters the inflammatory phenotype of macrophages by activating PPARs
Source: Acta Neuropathol Commun. 2013 Aug 2;1:43. doi: 10.1186/2051-5960-1-43 (PMC3893408; doi:10.1186/2051-5960-1-43)

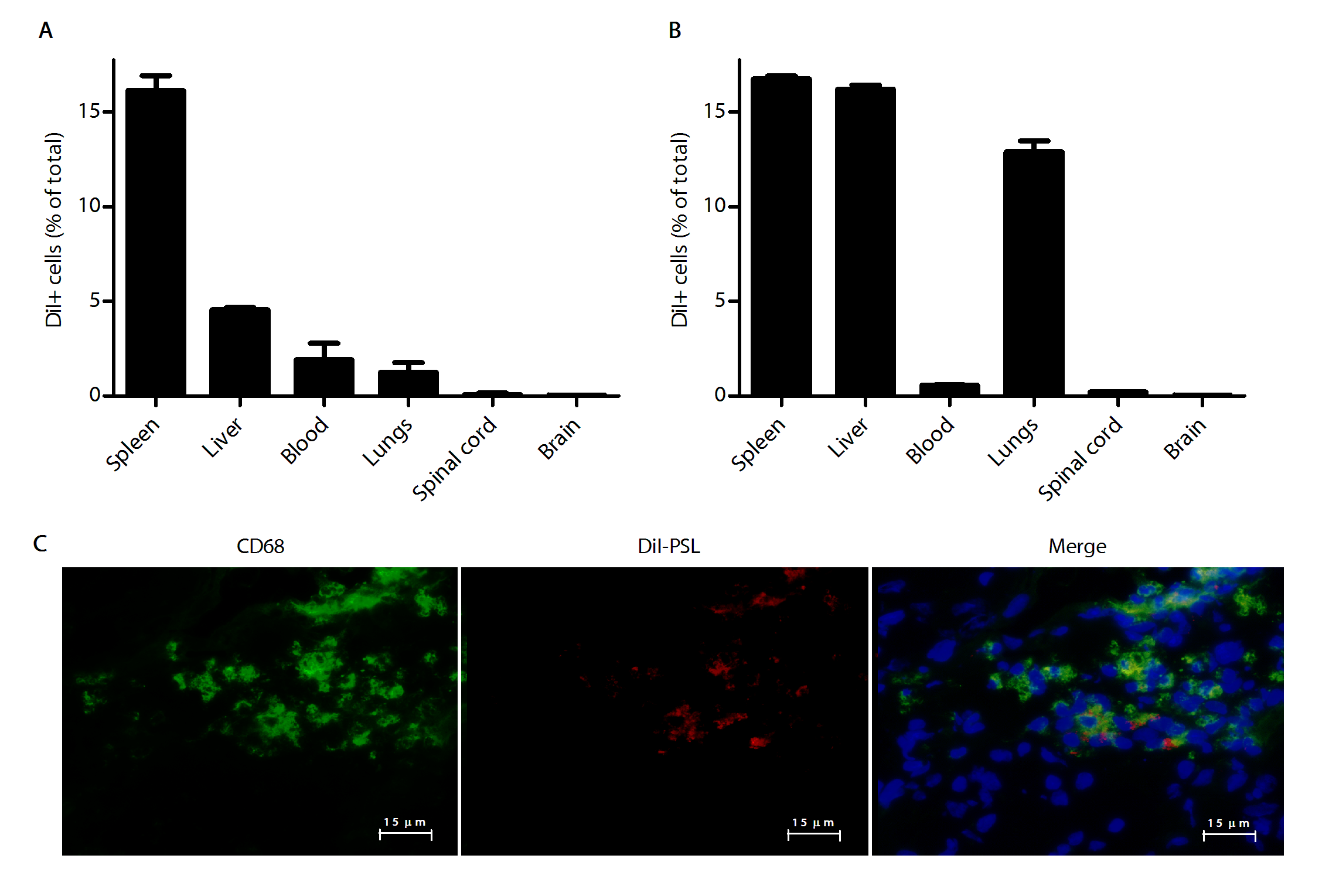

Supplement: Additional file 2: Figure S2 — Homing properties of liposomes following systemic administration. (a, b) Healthy (a) and immunized (b) rats were injected with 5 mg/kg DiI-labeled PSLs. Immunized animals received DiI-labeled liposomes one day after disease onset. After 24 h animals were sacrificed and the homing properties of liposomes were assessed by flow cytometry. To determine the homing of liposomes the amount of DiI+ cells (cells which internalized liposomes) was divided by the total number of organ cells analyzed. One representative experiment is shown (n=2). (c) Immunized rats were injected with 5 mg/kg DiI-labeled PSLs (DiI-PSL) at disease onset. After 24 h animals were sacrificed and spinal cord cryosections were stained with CD68 (40× magnification). One representative experiment is shown (n=3). [file 2051-5960-1-43-S2.tiff]

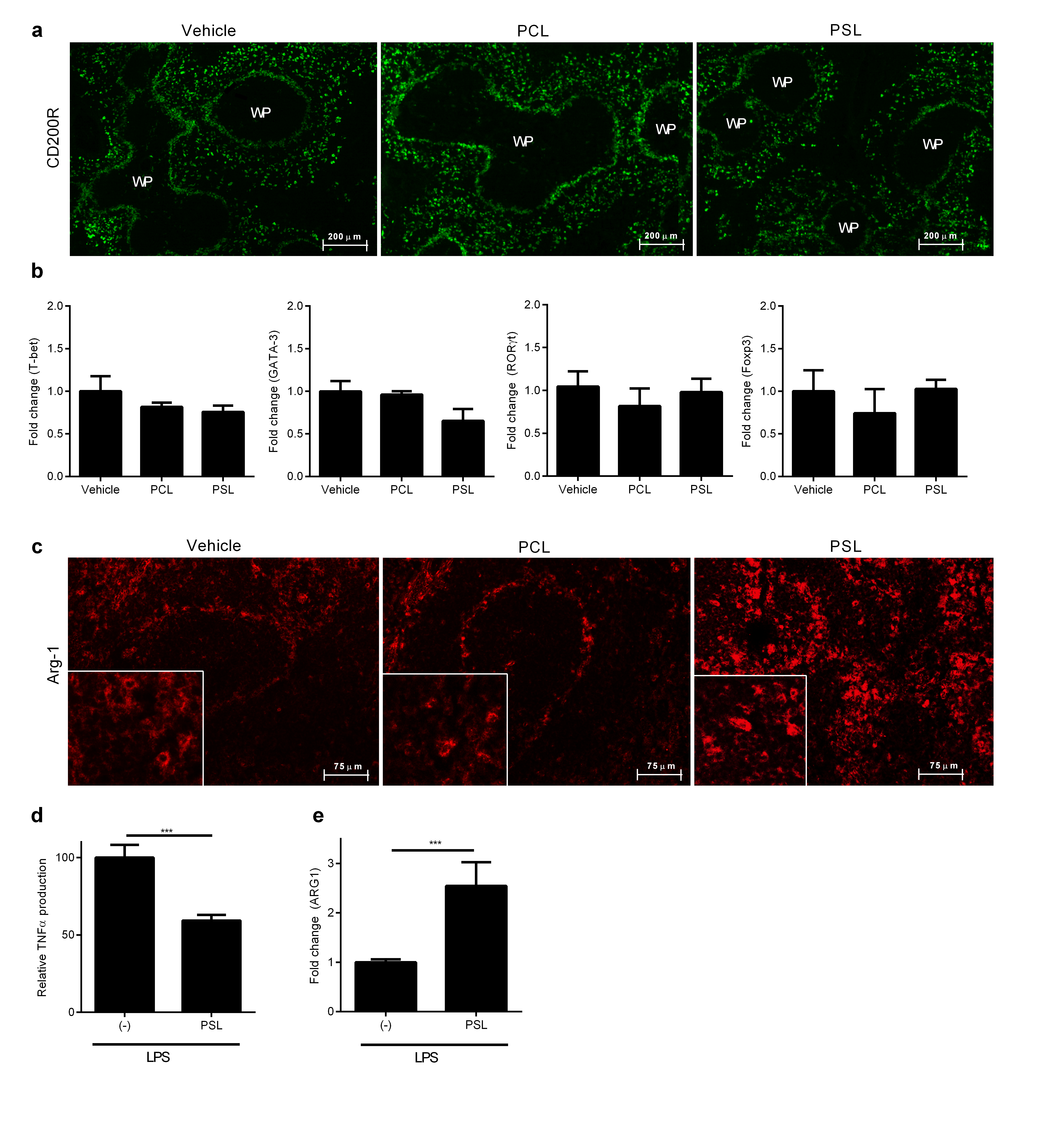

Supplement: Additional file 3: Figure S3 — Impact of liposomes on the inflammatory properties of splenic tissue in EAE animals. (a) Spleen tissue was isolated 10 dpi and stained with CD200R (10× magnification). One representative image is shown. WP: white pulp. (b) Comparison of fold changes between vehicle, PCL and PSL-treated spleens 10 dpi. Relative quantification of T-bet, GATA-3, RORγt and Foxp3 was accomplished by using the comparative Ct method. Data were normalized to the most stable reference genes, determined by Genorm (Pgk1 and Rpl13a). Data represent the mean ± SEM of 4 animals. (c) Spleen tissue was isolated 10 dpi and stained with ARG-1 (10× magnification). An identical exposure time and gain was used for all images. One representative image is shown. (d) Relative TNFα concentration in supernatants of LPS stimulated control and PSL-treated macrophages. The relative TNFα production is defined as the production of TNFα in experimental cultures divided by values in stimulated control cultures. Data represent the mean ± SEM of five independent experiments. (e) Comparison of fold changes between LPS stimulated control and PSL-treated macrophages. Relative quantification of ARG-1 gene expression was accomplished by using the comparative Ct method. Data were normalized to the most stable reference genes, determined by Genorm (TBP and HMBS). Data represent the mean ± SEM of three independent experiments. [file 2051-5960-1-43-S3.tiff]
