# Supplementary material for: Myelin alters the inflammatory phenotype of macrophages by activating PPARs
Source: Acta Neuropathol Commun. 2013 Aug 2;1:43. doi: 10.1186/2051-5960-1-43 (PMC3893408; doi:10.1186/2051-5960-1-43)
Supplement: Additional file 4: Table S1 — Primer details. [file 2051-5960-1-43-S4.docx]

**Supplementary Table 1:** Primer details.

| Gene symbol | Gene name | Forward and reverse primer |
| --- | --- | --- |
| Rat primers | |  |
| GAPDH  18S  CycA  ActB | Glyceraldehyde-3-phosphate dehydrogenase  18S subunit ribosomal RNA  Cyclophilin A  Beta actin | F: ACCACAGTCCATGCCATCAC  R: TCCACCACCCTGTTGCTGTA  F: ACGGACCAGAGCGAAAGCAT  R: TGTCAATCCTGTCCGTGTCC  F: TATCTGCACTGCCAAGACTGAGTG  R: CTTCTTGCTGGTCTTGCCATTCC  F: TGTCACCAACTGGGACGATA  R: GGGGTGTTGAAGGTCTCAAA |
| YWHAZ | Tyrosine 3-monooxygenase/tryptophan  5-monooxygenase activation | F: GATGAAGCCATTGCTGAACTTG  R: GTCTCCTTGGGTATCCGATGTC |
| Tbp  Rpl13A  HMBS  Pgk1 | TATA box binding protein  Ribosomal protein L13A  Hydroxymethyl-bilane synthase  Phosphoglycerate kinase 1 | F: TGGGATTGTACCACAGCTCCA  R: CTCATGATGACTGCAGCAAACC  F: GGATCCCTCCACCCTATGACA  R: CTGGTACTTCCACCCGACCTC  F: TCCTGGCTTTACCATTGGAG  R: TGAATTCCAGGTGAGGGAAC  F: ATGCAAAGACTGGCCAAG  R: AGCCACAGCCTCAGCATATTTC |
| iNOS | Inducible nitric oxide synthase 2 | F: GCATCCCAAGTACGAGTGGT |
|  |  | R: TGTTGTAGCGCTGTGTGTCA |
| TNFα | Tumor necrosis factor alpha | F: CTTATCTACTCCCAGGTTCTCTTCAA |
|  |  | R: GAGACTCCTCCCAGGTACATGG |
| ARG-1 | Arginase 1 | F: CAAGCTGGGAATTGGCAAAG |
|  |  | R: GGTCCAGTCCATCAACATCAAA |
| T-bet | T-box transcription factor | F: TCCTGTCTCCAGCCGTTTCT |
|  |  | R: CGCTCACTGCTCGGAACTC |
| GATA-3 | GATA binding protein 3 | F: ACCACGTCCCGTCCTACTAC |
|  |  | R: AGAGATCCGTGCAGCAGAG |
| RORγt | RAR-related orphan receptor gamma | F: ATCAATGCCAACCGTCCTGG |
|  |  | R: TGGAGGTGCTGGAAGTCCTGTAG |
| Foxp3 | Forkhead box P3 | F: CCCAGGAAAGACAGCAACCTT |
|  |  | R: CTGCTTGGCAGTGCTTGAGAA |
|  |  |  |
| *Human primers* |  |  |
| GAPDH  Pgk1  CycA  ActB  YWHAZ  Tbp  Rpl13A  HMBS | Glyceraldehyde-3-phosphate dehydrogenase  Phosphoglycerate kinase 1  Cyclophilin A  Beta actin  Tyrosine 3-monooxygenase/tryptophan  5-monooxygenase activation  TATA box binding protein  Ribosomal protein L13A  Hydroxymethyl-bilane synthase | F: GAGTCAACGGATTTGGTCGT  R: GACAAGCTTCCCGTTCTCAG  F: CTGGGCAAGGATGTTCTGTT  R: GCATCTTTTCCCTTCCCTTC  F: AGACTGAGTGGTTGGATGGC  R: TCGAGTTGTCCACAGTCAGC  F: GATCATTGCTCCTCCTGAGC  R: AAAGCCATGCCAATCTCATC  F: CTTGACATTGTGGACATCGG  R: TATTTGTGGGACAGCATGGA  F: TATAATCCCAAGCGGTTTGC  R: GCTGGAAAACCCAACTTCTG  F: AAGTTGAAGTACCTGGCTTTCC  R: GCCGTCAAACACCTTGAGAC  F: GAATGAAGTGGACCTGGTTGT  R: CTGGTTCCCACCACACTCTT |
| ADRP | Adipose differentiation related protein | F: TGTGAGATGGCAGAGAACGGT |
|  |  | R: CTGCTCACGAGCTGCATCATC |
| CTP1a | Carnitine palmitoyltransferase 1a | F: CTCAGTGGGAGCGGATGTTTA |
|  |  | R: TCGATGGTACACGACGATGTG |
| PDK4 | Pyruvate dehydrogenase kinase isozyme 4 | F: CCCGAGAGGTGGAGCATTT |
|  |  | R: GCATTTTCTGAACCAAAGTCCAGTA |
|  |  |  |
