# Supplementary material for: Myelin alters the inflammatory phenotype of macrophages by activating PPARs
Source: Acta Neuropathol Commun. 2013 Aug 2;1:43. doi: 10.1186/2051-5960-1-43 (PMC3893408; doi:10.1186/2051-5960-1-43)
Supplement: Additional file 5: Table S2 — Clinical data of MS patients and non-neurological controls. [file 2051-5960-1-43-S5.docx]

**Supplementary Table 2:** Clinical data of MS patients and non-neurological controls.

| Case | Age | Type of  MS | Sex | Post-morten delay (h:min) | | Disease  duration |
| --- | --- | --- | --- | --- | --- | --- |
|  | |  |  | |  |  |
| MS1  MS2  MS3  MS4 | 66  51  48  41 | SP  SP  SP  SP | F  M  F  F | | 6:00  11:00  8:10  8.25 | 23  15  9  11 |
| MS5 | 53 | SP | F | | 10:45 | 23 |
| MS6  Ctrl 1  Ctrl 2  Ctrl 3  Ctrl 4  Ctrl 5 | 66  70  64  68  68  69 | SP  N/A  N/A  N/A  N/A  N/A | F  M  F  F  M  F | | 6:00  7:30  8:35    5:45  10:10  8:30 | 23  N/A  N/A  N/A  N/A  N/A |
|  |  |  |  | |  |  |
